# LncRNA-HOTAIR activates autophagy and promotes the imatinib resistance of gastrointestinal stromal tumor cells through a mechanism involving the miR-130a/*ATG2B* pathway

**DOI:** 10.1038/s41419-021-03650-7

**Published:** 2021-04-06

**Authors:** Jinyan Zhang, Ke Chen, Yuexiao Tang, Xiaorui Luan, Xiaoxiao Zheng, Xuemei Lu, Jiayan Mao, Liqiang Hu, Shufen Zhang, Xianning Zhang, Wei Chen

**Affiliations:** 1 Department of Genetics, Zhejiang Provincial Key Laboratory of Genetic and Developmental Disorders, Institute of Cell Biology, Zhejiang University School of Medicine, Hangzhou, 310058 Zhejiang China; 2 Cancer Institute of Integrated Traditional Chinese and Western Medicine, Key Laboratory of Cancer Prevention and Therapy Combining Traditional Chinese and Western Medicine of Zhejiang Province, Zhejiang Academy of Traditional Chinese Medicine, Tongde Hospital of Zhejiang Province, Hangzhou, 310012 Zhejiang China; 3 Department of General Surgery, Sir Run Run Shaw Hospital, Zhejiang University School of Medicine, Hangzhou, 310016 Zhejiang China

**Keywords:** Oncogenes, Macroautophagy

## Abstract

Gastrointestinal stromal tumors (GISTs) are common neoplasms of the gastrointestinal tract that can be treated successfully using C-kit target therapy and surgery; however, imatinib chemoresistance is a major barrier to success in therapy. The present study aimed to discover alternative pathways in imatinib-resistant GISTs. Long noncoding RNAs (lncRNAs) are newly discovered regulators of chemoresistance. Previously, we showed that the lncRNA HOTAIR was upregulated in recurrent GISTs. In this study, we analyzed differentially expressed lncRNAs after imatinib treatment and found that HOTAIR displayed the largest increase. The distribution of HOTAIR in GISTs was shifted from nucleus to cytoplasm after imatinib treatments. The expression of HOTAIR was validated as related to drug sensitivity through Cell Counting Kit-8 assays. Moreover, HOTAIR was associated strongly with cell autophagy and regulated drug sensitivity via autophagy. Mechanistically, HOTAIR correlated negatively with miRNA-130a in GISTs. The downregulation of miRNA-130a reversed HOTAIR-small interfering RNA-induced suppression of autophagy and imatinib sensitivity. We identified autophagy-related protein 2 homolog B (*ATG2B)* as a downstream target of miR-130a and HOTAIR. *ATG2B* downregulation reversed the effect of pEX-3-HOTAIR/miR-130a inhibitor on imatinib sensitivity. Finally, HOTAIR was shown to influence the autophagy and imatinib sensitivity of GIST cells in mouse tumor models. Our results suggested that HOTAIR targets the *ATG2B* inhibitor miR-130a to upregulate the level of cell autophagy so that promotes the imatinib resistance in GISTs.

## Introduction

Gastrointestinal stromal tumors (GISTs) are the most common mesenchymal neoplasms of the gastrointestinal tract whose incidence is increasing^1^. GISTs are frequently driven by activating mutations in the receptor tyrosine kinase encoding genes *KIT* (KIT proto-oncogene, receptor tyrosine kinase) or *PDGFRA* (platelet -derived growth factor receptor alpha)^2^. These mutations result in constitutive activation of KIT or PDGFRA-mediated ligand-independent activation and signaling^3,4^. GISTs are most commonly found in the stomach (60%), small intestine, jejunum, and ileum (30%), but they also occur in the duodenum (5%), rectum (2–3%), colon (1–2%), and in the esophagus (<1%)^5,6^.

Imatinib is a small-molecule inhibitor of tyrosine kinase signaling enzymes, such as Abelson tyrosine-protein kinase 1 (BCR-ABL), KIT, and PDGFR, and is approved worldwide to treat GISTs^3,7–9^. However, about 12–14% of patients are resistant to imatinib and 40% will develop resistance^10^. Previous clinical studies of imatinib have indicated that the location of mutations within the pathogenic kinase is a significant factor in both the treatment response and the development of imatinib resistance^8^. Unfortunately, secondary mutations and other mechanisms resulting in resistance to imatinib have emerged as a clinical challenge^9^. Thus, imatinib has not been sufficiently effective as a chemotherapeutic, and new strategies must be developed to manage imatinib resistance. Therefore, further study of potential mechanisms of imatinib resistance in GIST and other treatment options is needed.

Long noncoding RNAs (lncRNAs, length > 200 nucleotides) are transcribed pervasively from the genome, and have been shown to regulate basic biochemical and cellular processes, such as invasion, survival, drug resistance, and metastasis in preclinical studies of cancer^11,12^. LncRNAs have emerged as a novel family of master cancer regulators^13^. Our previous published study also found that many lncRNAs were differentially expressed in recurrent GIST tissue compared with that in primary GIST tissue^14^. Therefore, we were curious as to whether lncRNAs have a role in the imatinib sensitivity of GISTs. The human lncRNA HOTAIR (HOX transcript antisense intergenic RNA) is a 2364 bp RNA transcribed from a 6449 bp gene located on the antisense strand of the *HOXC* gene locus on chromosome 12 and is a prime example of an oncogenic trans-acting lncRNA^12,13^. Dysregulated HOTAIR expression and function have been reported in at least 24 types of solid tumors^12,13^. As mentioned, our published studies found that lncRNAs including HOTAIR were upregulated in recurrent GIST. We wonder to know if HOTAIR was involved in imatinib resistance, and the potential mechanism.

Research into autophagy has increased massively after its discovery won the 2016 Nobel Prize in Physiology or Medicine^15^. Indeed, Autophagy has been found to be implicated in various biological processes, including drug sensitivity^16^. In GISTs, there were also many published studies reporting that autophagy participated in imatinib resistance^4^. And lncRNAs also could regulate cancer cell autophagy including GISTs cells^14^. Considering the widespread implications of lncRNAs and autophagy, we were prompted to investigate the role of HOTAIR in regulating autophagy.

Accumulating research has established that many lncRNAs are associated with other non-coding RNAs, especially microRNAs (miRNAs), and it has been reported that HOTAIR correlates negatively with miRNA-130a levels in several cancers^17,18^. MiRNAs are also recognized as important regulators of cancer through their post-transcriptional modulation of protein synthesis by binding to complementary sequences of the 3′-untranslated region (UTR) of mRNAs and inhibiting translation^19,20^. Thus, lncRNAs might regulate the expression of certain mRNAs by competitive binding to miRNAs. In addition, previous research showed that miR-130a had binding site within the 3′-UTR of *ATG2B*^21^. Therefore, we hypothesized that HOTAIR increased the expression of *ATG2B* through binding to its translation inhibitor, miR-130a.

In the present study, we report that HOTAIR expression was upregulated after imatinib treatment and we also studied the biological functions of HOTAIR in drug sensitivity of GISTs in vivo and in vitro. Moreover, mechanistic analysis revealed that HOTAIR might function as a competing endogenous RNA (ceRNA) that regulates the levels of miR-130a-3p. Our results showed a negative correlation between HOTAIR and miR-130a levels and between miR-130a and *ATG2B* levels, revealed that HOTAIR promotes imatinib resistance through regulating miR-130a/ATG2B, provided new pathways to overcome imatinib resistance in GISTs.

## Materials and methods

### Cell lines

The human GIST cell lines, GIST-882 and GIST-T1, were purchased from the Cosmo Bio, Co., Ltd., (Tokyo, Japan) and were not passaged for longer than 6 months continuously. GIST cells were cultured in Dulbecco’s modified Eagle’s medium (DMEM) (Gibco; Thermo Fisher Scientific, Inc., Waltham, MA, USA). All culture media were supplemented with 10% heat-inactivated fetal bovine serum (Gibco; Thermo Fisher Scientific, Inc.), 100 U/mL penicillin, and 100 mg/mL streptomycin. All cells were maintained at 37 °C in a humidified incubator with 5% CO_2_

### Cell transfection

The HOTAIR small interfering RNA (siRNA), *ATG2B* siRNA, pEX-3- HOTAIR, and the Negative Control siRNA were purchased from RiboBio (Guangzhou, China). Hsa-miRNA-130a mimic, the negative control mimic, and the hsa-miRNA-130a inhibitor and its negative control inhibitor were purchased from Genepharma (Shanghai, China). Cells were plated out in six-well plates at a density of 2 × 10^5^ per well. Transfections were performed using Lipofectamine 2000 (Invitrogen, Waltham, MA, USA) according to the manufacturer’s instructions.

### RNA extraction and quantitative real-time reverse transcription PCR (qRT-PCR)

Total RNA was extracted from cell lines using TRIzol (Invitrogen). cDNA was synthesized from the total RNA using the PrimeScript RT reagent Kit (Takara, Shiga, Japan). The relative mRNA levels were quantified by quantitative real-time PCR (qPCR) on a 480 II instrument (Roche Diagnostics, Basel, Switzerland) using the cDNA as a template with specific primers and TB Green qPCR Master Mix (Takara Bio, Inc.). The relative changes in the expression levels of mRNAs, miRNAs, and lncRNAs were normalized to that of *ACTB* (encoding β-actin) or U6 using the comparative 2^−ΔΔCt^ method^22^.

### Western blotting analysis

Cultured GIST cells after different treatments were harvested and total proteins were isolated using lysis buffer (Catalog No. 3868S, Cell Signaling Technology, Inc., Danvers, MA, USA). Following protein extraction, about 15 µg protein was loaded per lane, and separated using 10% sodium dodecyl sulfate-polyacrylamide gel electrophoresis. The proteins were then transferred onto polyvinylidene fluoride membranes (Millipore, Bedford, MA, USA) using electroblotting. The membranes were incubated with primary antibodies, including anti-Beclin1 (Catalog No. 3495P), anti-p62 (Catalog No. 8025S), anti-microtubule-associated protein 1 light chain 3 beta (Catalog No. 3868S) (all Cell Signaling Technology), and anti-*ATG2B* (Cat. No. ab189934) (Abcam, Cambridge, MA, USA). Membranes were then incubated with horseradish peroxidase-conjugated secondary antibodies (Beyotime Institute of Biotechnology, Jiangsu, China). The immunoreactive protein bands were visualized using chemiluminescence with an EZ-ECL Chemiluminescence Detection Kit (Biological Industries, Beit HaEmek, Israel). β-actin was detected using antibodies from Sigma (St. Louis, MO, USA; Cat. No. A1978) as the loading control.

### RNA fluorescence in situ hybridization (RNA-FISH)

RNA-FISH was performed with HOTAIR-specific probes (Genepharma, Shanghai, China). Cells were firstly fixed with 4% paraformaldehyde for 15 min and then processed step by step following the manufacturer’s instructions. Finally, cells were incubated with probes in darkness overnight at 37 °C. After that, the cells were washed with 0.1% Tween 20 pre-warmed at 42 °C for 5 min, then washed with 2 × SSC and 1 × SSC in the same way. Finally, cells were stained with DAPI for 20 min and imaged with a laser scanning confocal microscope (LSM 800, Zeiss, Oberkochen, Germany).

### Cell Counting kit-8 (CCK-8) assay

GIST cells were seed at a density of 6 × 10^3^ cells per well in 96-well microplates in medium. After incubation overnight, the cells were treated with different imatinib (Selleck, Houston, TX, USA; S2475) concentrations (0, 10, 20, 40, 60, and 80 µmol/L) for 48 h. Then, to measure cell viability, 10 μL CCK-8 reagent (Catalog No. AD10, Dojindo Molecular Technologies, Inc., Kumamoto, Japan) was mixed with 90 μL of DMEM and was used to replace the complete media to add to each well. After incubation for 30 min, the absorbance value at 450 nm was measured using a microplate reader (Thermo Fisher Scientific).

In experiments in combination with autophagy-regulating drugs, the cells should be treated with single 0.5 mM 3-methyladenine (3-MA) (Selleck, USA; S2767) or 10 nM Rapa (Selleck, USA; S1039) for about 3 h in advance. Then treat with imatinib in the presence of corresponding autophagy-regulating drugs.

### Apoptosis analysis

GIST cells or transfected cells were seeded into six-well plates at a density of 1 × 10^5^ cells per well and incubated with 20 μmol/L imatinib for 24 h. Then, cells were stained with 5 μL of Annexin V-fluorescein isothiocyanate and 5 μL propidium iodide (Abcam) in the dark for 30 min at 25 °C. Finally, results were obtained with a FACS Caliber flow cytometer (BD, San Jose, CA, USA).

### Autophagic flux assay

Cells were established to stabilize mRFP-GFP-LC3 (Hanbio, Shanghai, China). The cells were subjected to different treatments for 48 h. Then, after washing three times with phosphate-buffered saline (PBS), the cells were fixed with 4% paraformaldehyde for 30 min at 25 °C. Images were captured from 10 to 15 fields of per sample using a laser scanning confocal microscope (LSM 800, Zeiss, Oberkochen, Germany).

### Luciferase reporter assay

The target sequences (human wild-type HOTAIR (h-HOTAIR-WT), human mutated HOTAIR, human wild-type *ATG2B* (h-*ATG2B*-WT), and human mutated *ATG2B* (h-*ATG2B*-MUT)) were cloned into the pmiR-RB-Report^TM^ vector containing the firefly luciferase reporter gene (Ribobio Co., Guangzhou, China). For the reporter assays, 1 × 10^4^ human embryonic kidney cells (HEK293T) cells were cotransfected with each plasmid, together with 50 nM miR-130a or its Negative Control, according to the manufacturer’s instructions. After 48 h, relative Renilla luciferase activity (firefly luciferase/Renilla luciferase) was measured using the Dual-Luciferase reporter assay.

### Transmission electron microscopy

After transfection with siHOTAIR/pEX-3-HOTAIR, GIST cells were treated with imatinib (10 μM) overnight. Then, cells were collected and fixed in 2.5% glutaraldehyde at 4 °C for 4 h. Following treatment with 1% osmic acid for 1 h at room temperature, then rinsed with 0.1 M phosphate buffer, dehydrated by stepwise incubation in ethanol (ethanol concentration gradient: 30%, 50%, 70%, 90%, 100%), and then embedded in EPON812 resin (Structure Probe, Inc.) according to the manufacturer’s instructions. The samples were sectioned at 50–60 nm, stained with 3% uranyl acetate plus lead citrate, and images were captured using a transmission electron microscope (magnification, ×25,000), and five fields of view per sample were analyzed.

### In vivo analysis

Male nude mice (aged about 4 weeks; weight about 20 g) were bought from Silaike Experimental Animal Center (Shanghai, China). GIST-T1 cells (1 × 10^6^) in 100 μL of PBS were injected into the flanks of nude mice to establish a mouse tumor xenograft model. After successful tumor formation, mice were randomized into four groups (*n* = 8/group) and then injected with buffer, imatinib (2 mg/100 μL in PBS), siHOTAIR (2 nmol/100 μL in PBS), or imatinib and siHOTAIR, every other day during the 14 days. During the experiment, the mice body weights and tumor volumes were recorded in real time. After completing all treatments and sacrificing the mice, the tumors were excised for further study.

Ki-67 immunohistochemical staining and terminal deoxynucleotidyl transferase nick-end-labeling (TUNEL) assays were applied, respectively, to assess the tumor cell proliferation and apoptosis. Moreover, immunohistochemistry for p62 was performed to detect the level of autophagy in tumor cells.

### Statistical analysis

All statistical analyses were performed using GraphPad Prism 7 (GraphPad Software, Inc., La Jolla, CA, USA). Data are presented as means ± SD from three independent experiments. Two-tailed Student’s *t*-test and one-way ANOVA were used to determine statistical differences between two groups and more than two groups, respectively. *P* < 0.05 was considered statistically significant.

## Results

### HOTAIR is upregulated in recurrent GISTs tissue and is associated with drug sensitivity to imatinib

**Fig. 1:**
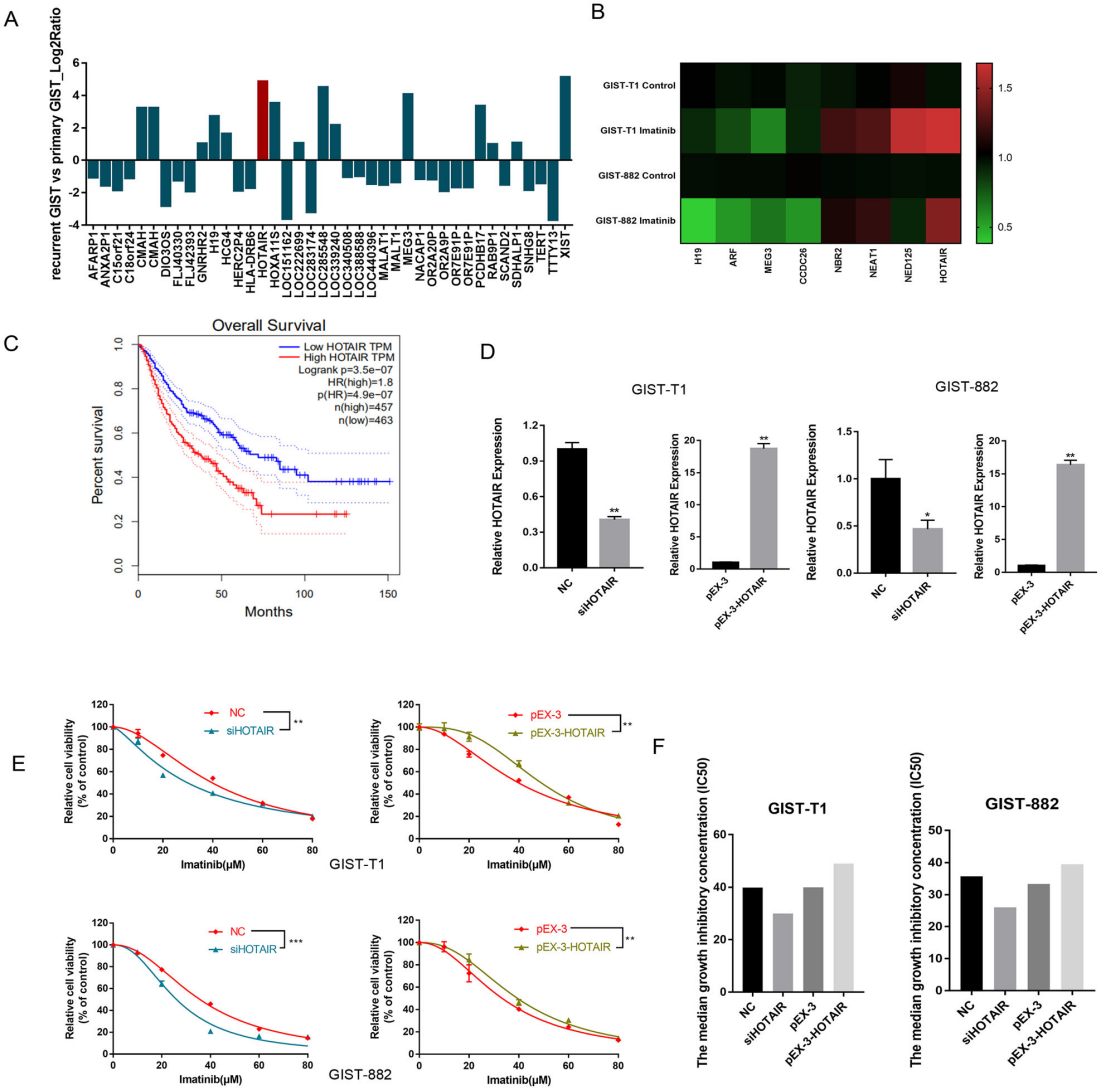
HOTAIR is upregulated in recurrent GIST tissue and is associated with imatinib sensitivity. **A** Some of the lncRNAs that were differentially expressed in recurrent GIST tissues compared with that in primary GIST tissues. **B** Heat map of differentially expressed lncRNAs after imatinib treatment. **C** Overall survival based on the high expression of HOTAIR in several kinds of gastrointestinal tumors. **D** The change in HOTAIR expression after transfection, as assessed using qRT-PCR in GIST-T1 and GIST-882 cells. *P < 0.05, **P < 0.01 vs. NC or pEX-3. **E**, **F** CCK-8 assays were performed to examine imatinib sensitivity of GIST cells, and the IC50 values are plotted as bar graphs. *P < 0.05, **P < 0.01 vs. NC.

Our previous published study observed that many lncRNAs were differentially expressed in recurrent GIST tissue compared with that in primary GIST tissue^14^. The expression level of selected lncRNAs, including HOTAIR, is shown in Fig. 1A. To further explore the potential functions of lncRNAs in drug sensitivity in GISTs, we carried out lncRNA expression profiling on RNA samples of imatinib-treated and -untreated cells (Fig. 1B). Among the eight lncRNAs tested, HOTAIR displayed the largest increase after imatinib treatment (Table 1). Furthermore, although there is no lncRNA expression database for GISTs, the research significance of HOTAIR was confirmed by the overall survival curves obtained from the GEPIA database regarding the high expression of HOTAIR in several types of gastrointestinal tumors, including stomach adenocarcinoma, colon adenocarcinoma, esophageal carcinoma, rectal adenocarcinoma (Fig. 1C).

**Table 1:** Initial profiling of long noncoding RNAs in response to Imatinib treatment in gastrointestinal stromal tumors (GIST-T1/GIST-882).

	LncRNA	Fold change	
		GIST-T1	GIST-882
1	H19	0.9646	0.3858
2	ARF	0.7860	0.5648
3	MEG3	0.7750	0.6810
4	CCDC26	0.8801	0.6022
5	NBR2	1.2254	1.0836
6	NEAT1	1.3277	1.2259
7	NED125	1.4166	0.9557
8	HOTAIR	1.5777	1.4353

Next, we transfected GIST cells with either pEX-3-HOTAIR (to overexpress HOTAIR) or siHOTAIR (to silence HOTAIR) to evaluate whether HOTAIR regulates drug sensitivity. As shown in Fig. 1D, the expression of HOTAIR changed predictably after transfection. CCK-8 assays indicated that the upregulation of HOTAIR remarkably decreased the cytotoxicity of imatinib to GIST-T1 and GIST-882 cells, whereas the downregulation of HOTAIR had the opposite effects (Fig. 1E, F). These results demonstrated that HOTAIR could regulate the imatinib sensitivity of GISTs.

### HOTAIR regulates cell autophagy to sensitizes GIST cells imatinib

**Fig. 2:**
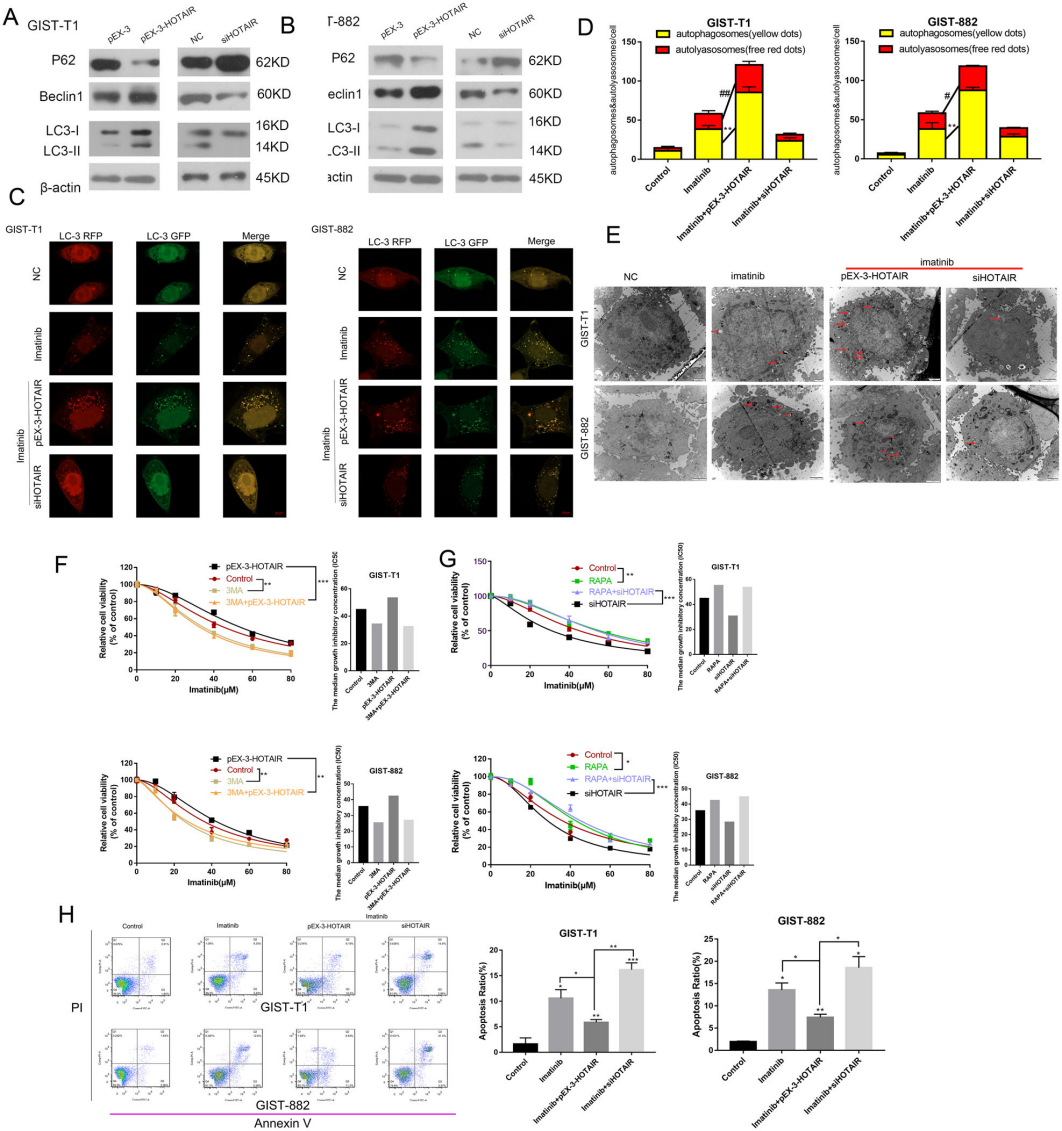
HOTAIR regulates cell autophagy. **A**, **B** Western blotting was performed to detect the levels of autophagy-related proteins in GIST cells after transfection. **C**, **D** Confocal microscopy: red dots indicate autolysosomes. ^#^*P* < 0.05, ^##^*P* < 0.01 vs. imatinib: yellow dots indicate autophagosomes. *P < 0.05, **P < 0.01 vs. imatinib. **E** Autophagosomes were observed by electron microscopy. **F** CCK-8 assays were performed after GIST cells were treated in four different ways (negative control, 3-MA, pEX-3-HOTAIR, and 3-MA plus pEX-3-HOTAIR). **P < 0.01, ***P < 0.001 vs. control or pEX-3-HOTAIR. **G** CCK-8 assays were performed after GIST cells were treated in four different ways (negative control, RAPA, siHOTAIR, RAPA plus siHOTAIR). *P < 0.05, **P < 0.01, ***P < 0.001 vs. control or siHOTAIR. **H** Flow cytometry analysis to determine the effect of HOTAIR in rescuing GIST cells from apoptosis. *P < 0.05, **P < 0.01, ***P < 0.001 vs. control or Imatinib + pEX-3-HOTAIR.

Previous studies have shown that autophagy, as a self-protection mechanism, affects the sensitivity of tumor cells to drugs. Therefore, we speculated that autophagy is involved in the drug resistance of GIST cells, and HOTAIR might function by regulating autophagy. First, we transfected cells with siHOTAIR or pEX-3-HOTAIR and analyzed the expression levels of autophagy-related proteins, including P62, Beclin1, and LC3I/II (Fig. 2A, B). Based on the results, we found that autophagy-associated proteins correlated positively with HOTAIR expression. Likewise, the images obtained using confocal microscopy showed that the numbers of autophagosomes increased markedly in cells transfected with pEX-3-HOTAIR, and were reduced in GIST cells transfected with siHOTAIR (Fig. 2C, D). What is more, electron microscopy results also showed that HOTAIR silencing decreased numbers of autophagosomes compared with the negative control group (Fig. 2E). These results indicated that HOTAIR might regulate cell autophagy.

To confirm that HOTAIR regulates cell sensitivity to imatinib via its effect on autophagy, we applied autophagy-regulating drugs, including an autophagy inhibitor, 3-MA, and an autophagy inducing agent, rapamycin (RAPA), to alter cell autophagy. The result showed that after treatment with 3-MA, transfection with pEX-3-HOTAIR no longer influenced drug sensitivity in GIST cells (Fig. 2F). Similarly, in the presence of RAPA, the HOTAIR siRNA also no longer influenced the drug sensitivity in GIST cells (Fig. 2G). Therefore, these results affirmed that HOTAIR regulates imatinib sensitivity in GIST cells by affecting cell autophagy.

Furthermore, flow cytometric analysis (Fig. 2H) showed that HOTAIR reduced imatinib-induced apoptosis in GIST cells. Thus, HOTAIR regulates cell autophagy in GIST-T1 and GIST-882 cells, thereby protecting them from apoptosis.

### HOTAIR levels correlate negatively with miRNA-130a levels in GISTs

**Fig. 3:**
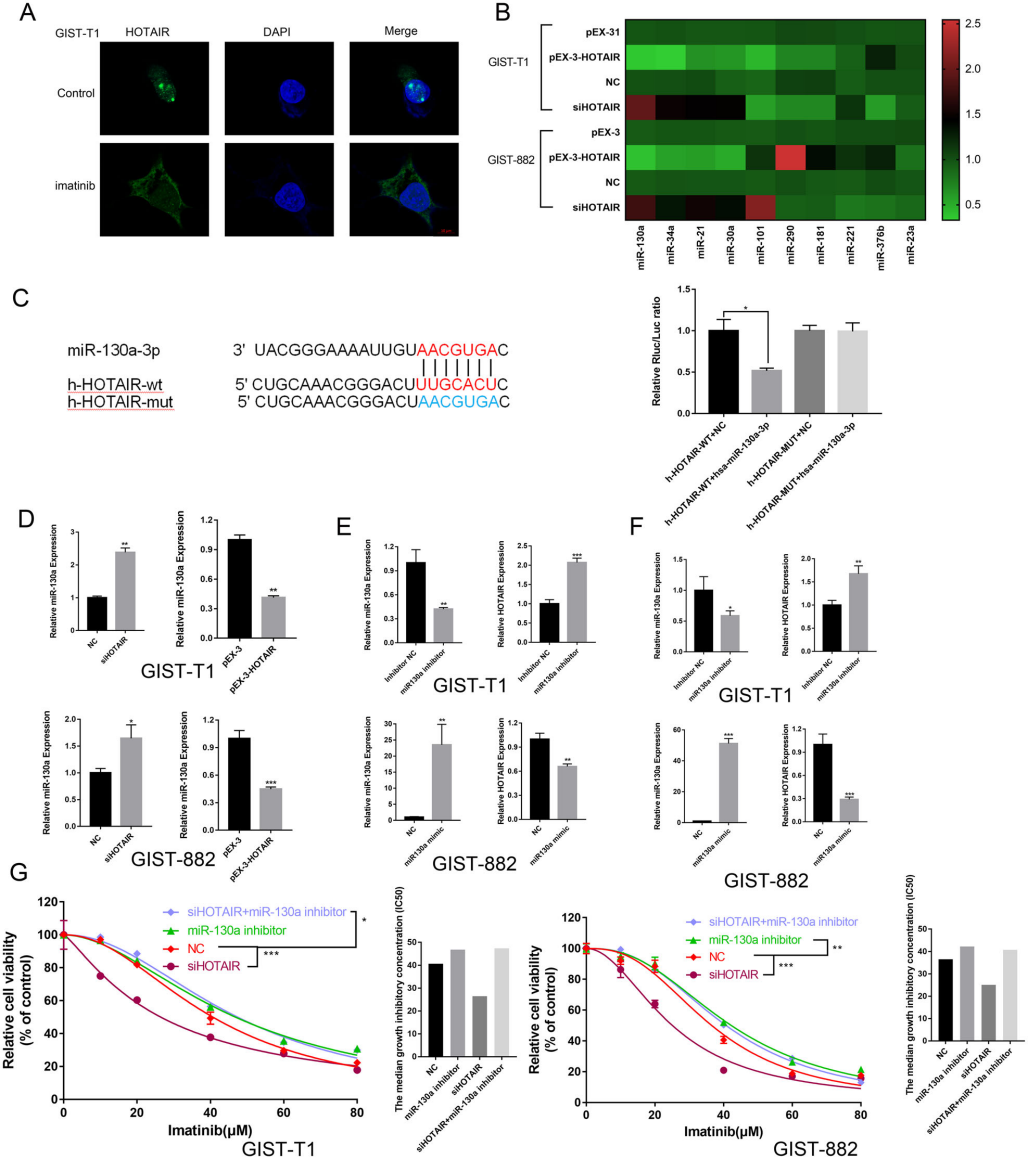
HOTAIR expression correlates negatively with miRNA-130a levels in GISTs. **A** The distribution of HOTAIR in GIST-T1. **B** Heat map of differentially expressed miRNAs after regulating the expression of HOTAIR. **C** Binding sites of HOTAIR in miRNA-130a-3p predicted using TargetScan and the results of Luciferase reporter gene assays in HEK293T cells. *P < 0.05 vs. h-HOTAIR-WT + NC. **D** Cells transfected with pEX-3-HOTAIR showed markedly decreased levels of miRNA-130a and siHOTAIR upregulated the levels of miRNA-130a in GIST cells. *P < 0.05; **P < 0.01; ***P < 0.001 vs. NC or pEX-3. **E** The efficiency of transfection of the miRNA-130a mimic or miRNA-130a inhibitor. **P < 0.01; ***P < 0.001 vs. NC or inhibitor NC. **F** The miRNA-130a mimic notably decreased the levels of HOTAIR and the miRNA-130a inhibitor increased the mRNA levels of HOTAIR in GIST cells. *P < 0.05; **P < 0.01; ***P < 0.001 vs. NC or inhibitor NC. **G** CCK-8 assays were used after co-transfection GIST cells with the miR-130a inhibitor and siHOTAIR. *P < 0.05; **P < 0.01; ***P < 0.001 vs. NC.

To investigate the mechanism by which HOTAIR regulates autophagy, we used RNA-FISH technology to find the localization of the HOTAIR, as shown in Fig. 3A HOTAIR was shifted from nucleus to cytoplasm after imatinib treatments in GIST-T1 cells. Sponging miRNAs as ceRNAs is one of the main ways by which cytoplasmic lncRNAs exert their functions. To investigate the mechanism by which HOTAIR regulates autophagy, a series of autophagy-related miRNAs were examined after HOTAIR silencing or overexpression. As shown in Fig. 3B, the levels of miRNAs involved in the regulation of cellular autophagy, such as miR-130a, miR-34a, miR-21, and miR-30a, were found to correlate negatively with HOTAIR expression; the specific values are listed in Table 2. Furthermore, the TargetScan (www.targetscan.org) result predicted that the expression of miR-130a is highly conserved and that miR-130a can bind to HOTAIR (Fig. 3C), suggesting that it could be regulated by HOTAIR. Furthermore, to determine if the level of miR-130a is regulated by HOTAIR because of the direct interaction between miR-130a and its predicted binding site in HOTAIR, we cotransfected HEK293T cells with HOTAIR luciferase reporter constructs and the miR-130a mimic (Fig. 3C). The luciferase reporter assays confirmed that miR-130a was a direct target of HOTAIR.

**Table 2:** Profiling of microRNAs after HOTAIR transfection in gastrointestinal stromal tumors (GIST-T1/GIST-882).

	MicroRNA	GIST-T1		GIST-882	
		pEX-3-HOTAIR	siHOTAIR	pEX-3-HOTAIR	siHOTAIR
1	miR-130a	0.3662	2.1098	0.3888	1.7591
2	miR-34a	0.3181	1.5206	0.5904	1.2899
3	miR-21	0.6517	1.3889	0.6211	1.5088
4	miR-30a	0.5867	1.3988	0.4858	1.4031

To further confirm that HOTAIR expression correlated negatively with miRNA-130a levels in GISTs, we first transfected GIST cells with either pEX-3-HOTAIR or siHOTAIR and then detected the differential expression of miRNA-130a. As shown in Fig. 1D, the efficiency of transfection of pEX-3-HOTAIR or siHOTAIR was excellent. The qRT-PCR results showed that in GIST cells the upregulation of HOTAIR reduced the miRNA-130a level and HOTAIR downregulation increased the miRNA-130a level (Fig. 3D).

We also transfected either the miRNA-130a mimic or the miRNA-130a inhibitor to determine their effect on HOTAIR levels (Fig. 3E, F). These data demonstrated that HOTAIR has a negative correlation with miRNA-130a levels in GIST cells.

Next, we determine whether HOTAIR improves drug sensitivity by regulating miR-130a levels in GIST cells. We cotransfected GIST cells with the miR-130a inhibitor and siHOTAIR, treated them with imatinib, and detected cell viability via a CCK-8 assay (Fig. 3G). The result indicated that downregulating HOTAIR did not further affect drug sensitivity after downregulating miR-130a compared with only downregulating miR-130a, suggesting that HOTAIR exerts its influence on imatinib sensitivity in GIST cells via its negative effect on miR-130a levels.

### HOTAIR regulates cell autophagy by targeting miR-130a

**Fig. 4:**
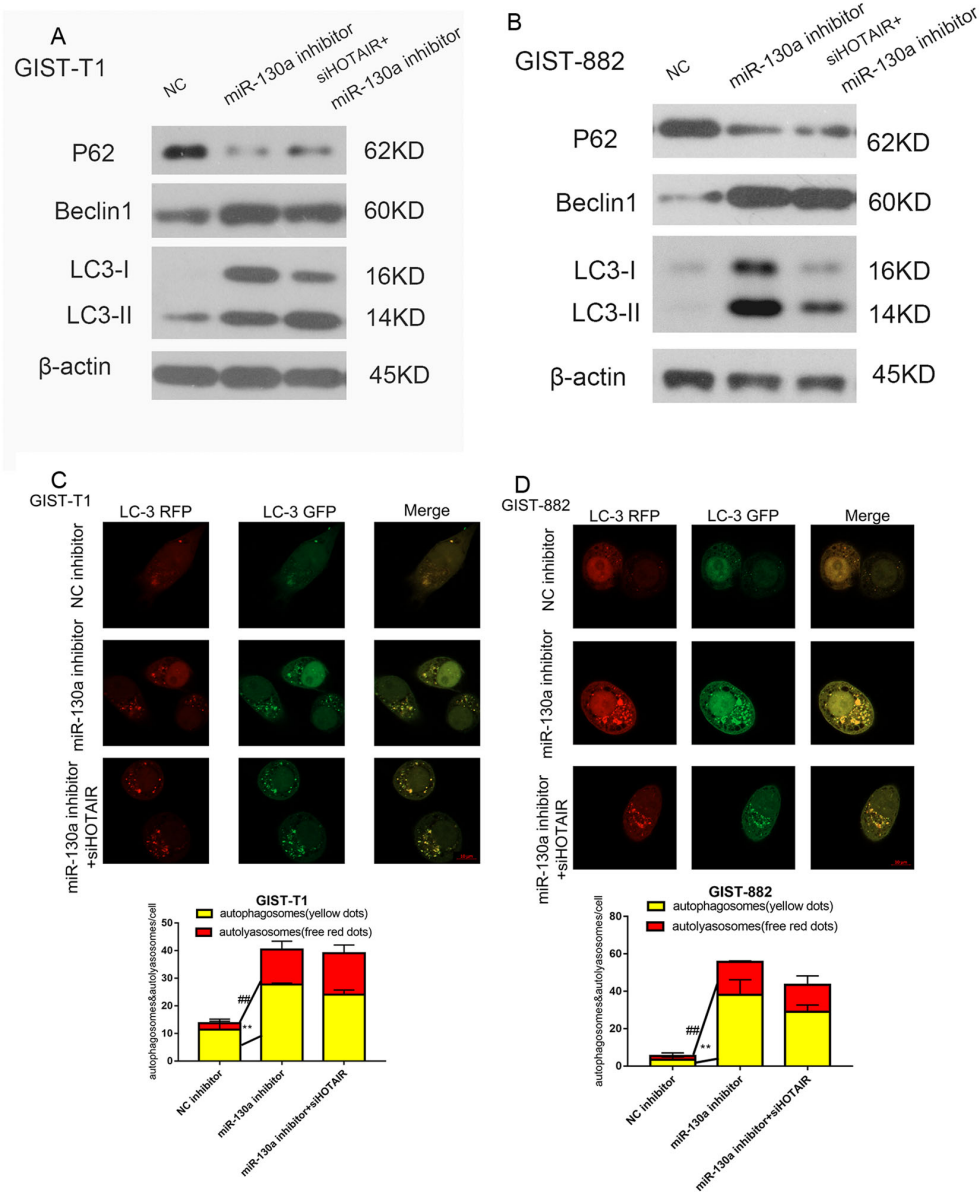
HOTAIR regulates cell autophagy via miR-130a. **A**, **B** The levels of autophagy-related proteins (Beclin1, p62, LC3) were detected using western blotting. **C**, **D** Confocal microscopy images: red dots indicate autolysosomes. ^##^*P* < 0.01 vs. the NC inhibitor; yellow dots indicate autophagosomes. **P < 0.01 vs. the NC inhibitor.

Next, we investigated the effects of the differential expression of HOTAIR and miR-130a on the regulation of cell autophagy. After cotransfecting GIST cells with the miR-130a inhibitor and siHOTAIR, the changes in the levels of autophagy-related proteins indicated increased levels of autophagy (Fig. 4A, B). Confocal laser scanning microscopy was then employed to detect autophagosome formation (Fig. 4C, D). Compared with the results shown in Fig. 2, the influence of siHOTAIR on autophagy was reversed by the miR-130a inhibitor. These results confirmed that HOTAIR regulates cell autophagy via miR-130a.

### In GIST cells, miR-130a directly targets *ATG2B*

**Fig. 5:**
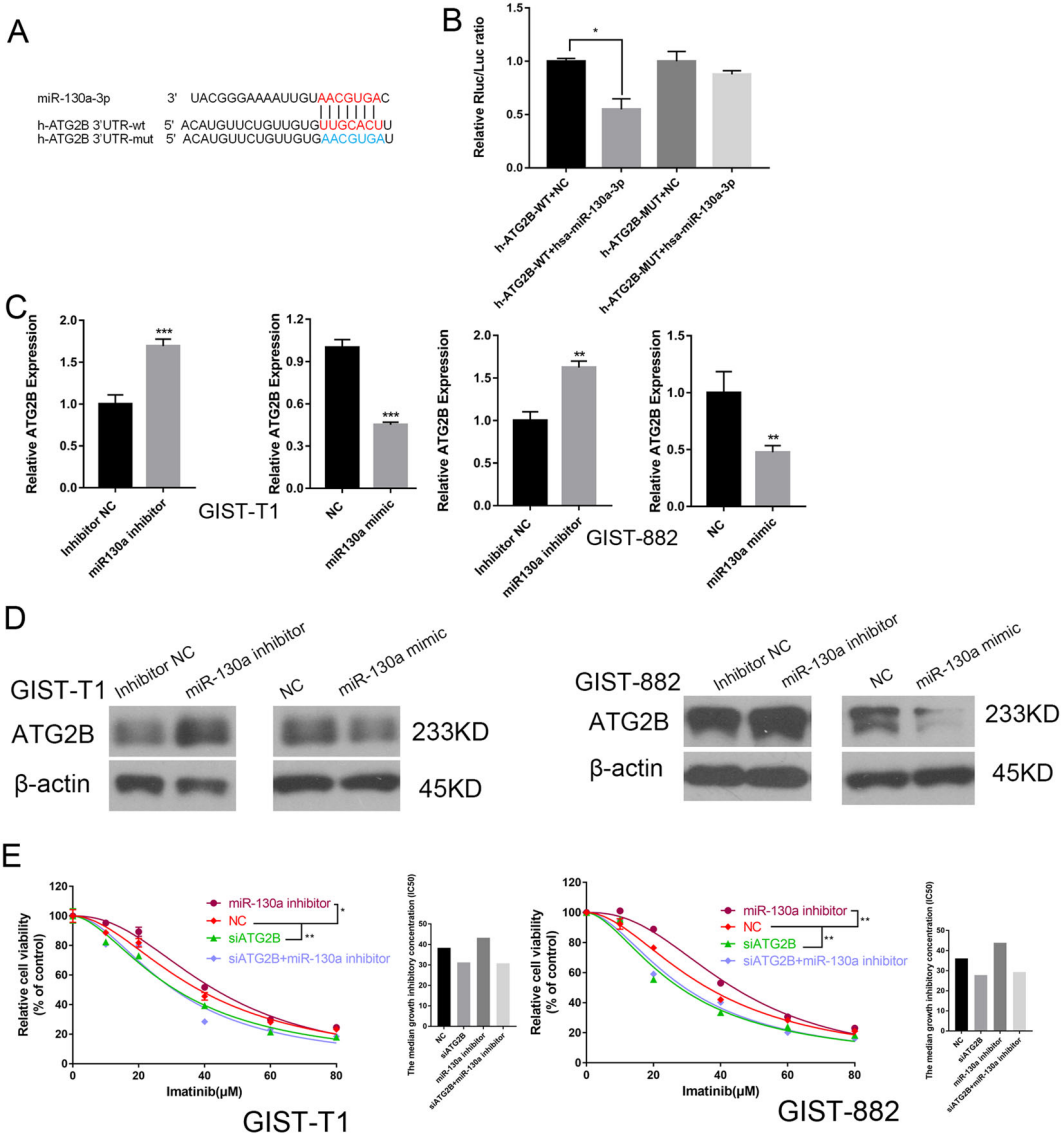
miR-130a directly targets *ATG2B*. **A** The TargetScan predicted the binding site for miR-130a in the 3′-UTR of *ATG2B*. **B** Dual-Luciferase reporter assays analyzing the putative targets of miR-130a. *P < 0.05 vs. h-*ATG2B*-WT + NC. **C**, **D** The results of western blotting and qRT-PCR showing that miR-130a regulates *ATG2B* in GIST cells. **P < 0.01; ***P < 0.001 vs. the NC or inhibitor NC. **E** CCK-8 assays were used to measure the changes in cell viability after different transfection of GIST cells. *P < 0.05; **P < 0.01 vs. NC.

Previously, it was reported that miR-130a targeted *ATG2B* and prevented autophagy in leukemia cells^21^. Thus, we hypothesized that miR-130a has the same effects on *ATG2B* in GIST cells. We identified a presumptive binding site for miR-130a in the 3′-UTR of *ATG2B* (Fig. 5A). Dual-Luciferase reporter assays were applied to conformed that miR-130a targeted and regulated *ATG2B* expression (Fig. 5B).

In addition, qRT-PCR and western blotting results demonstrated that the upregulation of miR-130a decreased the expression of *ATG2B* in GIST cells, while the downregulation of miR-130a increased *ATG2B* levels in GIST cells (Fig. 5C, D). Moreover, we cotransfected GIST cells with the miR-130a inhibitor and the *ATG2B* siRNA, and the results of a CCK-8 assay demonstrated that the downregulation of *ATG2B* increased the chemosensitivity of GIST cells to imatinib and the function of miR-130a is reversed after downregulating *ATG2B* (Fig. 5E). Thus, in the present study, we concluded that *ATG2B* has a correlationship with chemosensitivity to imatinib and miR-130a influences chemosensitivity to imatinib by regulating *ATG2B*.

### HOTAIR also regulates *ATG2B* expression in GISTs cells

**Fig. 6:**
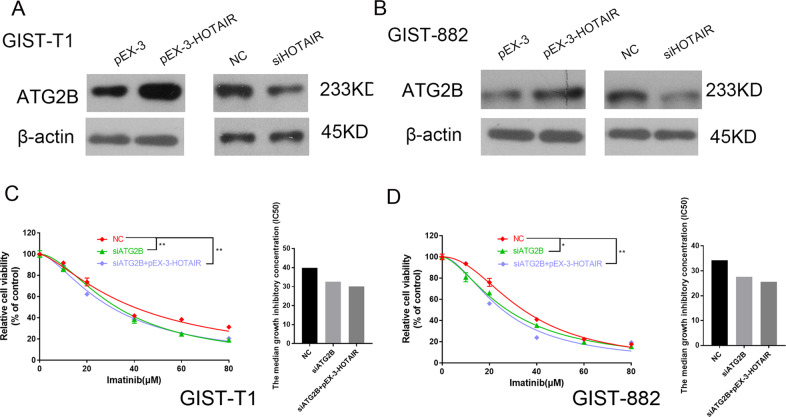
HOTAIR also regulates *ATG2B* levels. **A**, **B** The results of western blotting showing that HOTAIR regulates the expression of *ATG2B*. **C**, **D** The *ATG2B* siRNA or the *ATG2B* siRNA and the HOTAIR siRNA together were transfected into the GIST cells, and the resultant changes in cell viability after imatinib treatment were estimated through the CCK-8 assay. *P < 0.05; **P < 0.01 vs. NC.

Given that *ATG2B* is a downstream target of miR-130a and HOTAIR expression correlated negatively with miRNA-130a levels in GISTs cells, western blotting was performed to determine if HOTAIR also regulates *ATG2B* levels in GIST cells (Fig. 6A, B). We cotransfected GIST cells with the HOTAIR siRNA and *ATG2B* siRNA, and the changes in cell viability after imatinib treatment were estimated using CCK-8 assays. The CCK-8 assay results suggested that HOTAIR affects the sensitivity of GIST cells to imatinib in association with *ATG2B* (Fig. 6C, D). Taken together, we concluded that HOTAIR exerts its role on the chemosensitivity in GIST cells by regulating *ATG2B* via sponging miRNA-130a.

### The downregulation of HOTAIR increases imatinib sensitivity in GIST cells in vivo

**Fig. 7:**
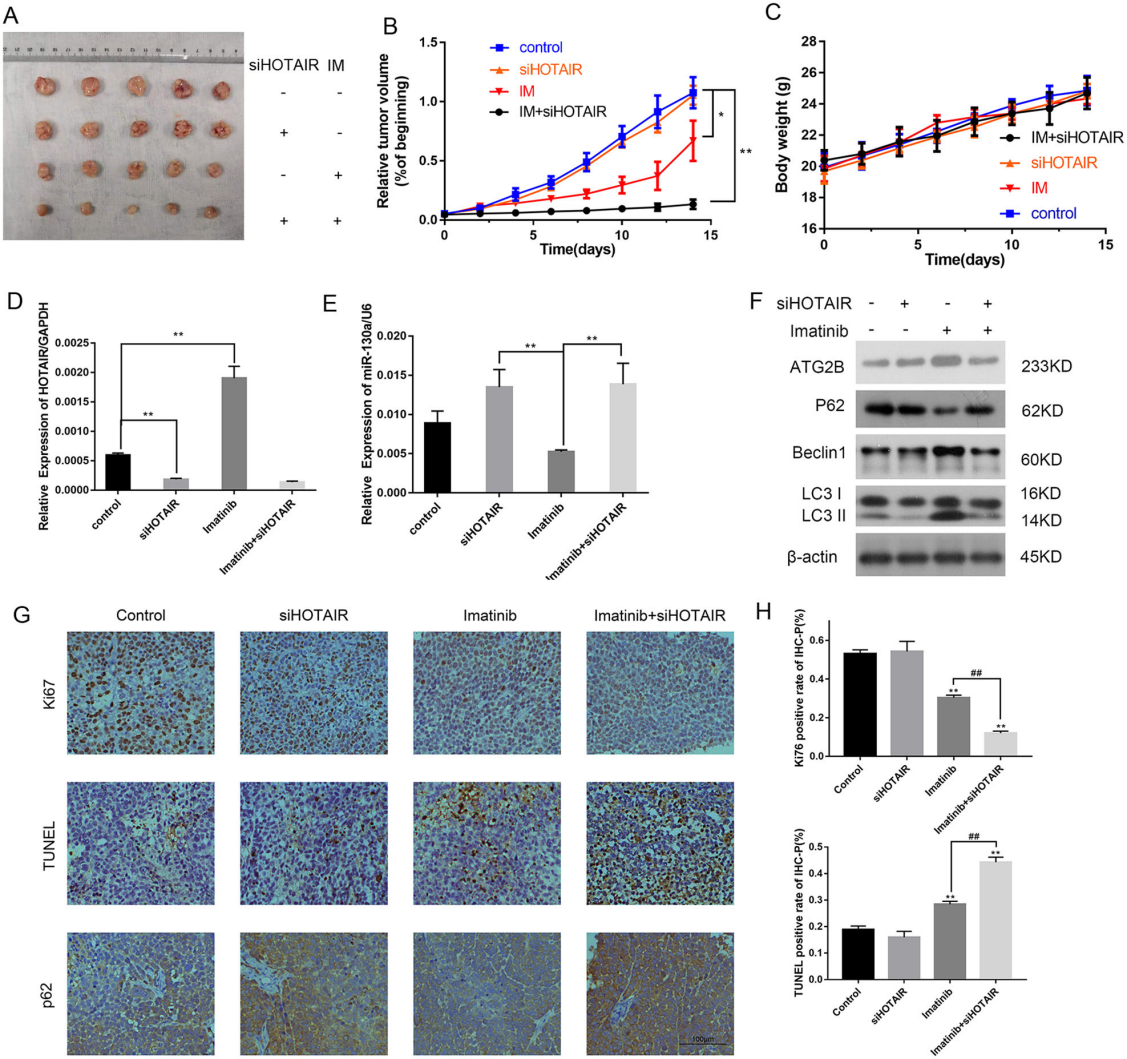
The downregulation of HOTAIR increased the sensitivity of GIST cells to imatinib in vivo. **A** Size of the excised tumors after 14 days of treatment with or without imatinib in the presence or absence of siHOTAIR. **B** Average percent change in tumor volume relative to the beginning for each treatment. *P < 0.05; **P < 0.01 vs. control. **C** Changes in body weight for each treatment. **D**, **E** The relative expression levels of HOTAIR and miR-130a were analyzed in the four groups using qRT-PCR. **P < 0.01 vs. control or imatinib. **F** The levels of autophagy-related proteins (*ATG2B*, Beclin1, p62, and LC3) were detected using western blotting. **G** Representative pictures of Ki-67 staining, TUNEL assays, and immunohistochemistry for p62. **H** The positive rates of Ki-67 staining and TUNEL assays. **P < 0.01 vs. control. ^##^*P* < 0.01 vs. imatinib.

To better investigate the effects of HOTAIR on drug resistance, in vivo studies were taken. GIST cells were injected into the flanks of nude mice to establish a mouse tumor xenograft model and then inject with buffer, imatinib, siHOTAIR, or imatinib and siHOTAIR. We observed that siHOTAIR had no obvious influence on tumor growth alone; however, combined with imatinib, downregulating HOTAIR remarkably inhibited tumor growth, indicating that knocking down HOTAIR enhanced the efficacy of imatinib (Fig. 7A, B). The body weight of the mice did not show a noticeable difference among the four groups, suggesting these treatments had no obvious toxicity in the mice (Fig. 7C).

Subsequently, the relative expression of HOTAIR and miR-130a was analyzed in four groups’ tumor samples using qRT-PCR (Fig. 7D, E), which demonstrated the successful intervention of HOTAIR expression by siHOTAIR in vivo. In addition, we analyzed the level of autophagy via western blotting and found that siHOTAIR suppressed IM-induced autophagy (Fig. 7F). Ki-67 staining, TUNEL assays, and immunohistochemistry for p62 were performed, which further verified that HOTAIR promoted proliferation and autophagy (Fig. 7G). The positive rates of Ki-67 staining and TUNEL assays are shown in Fig. 7H. Immunohistochemistry for p62 demonstrated the lightest staining in imatinib group, representing the lowest levels of p62, and the darkest staining was observed in the siHOTAIR and imatinib + siHOTAIR groups. Collectively, our findings indicated that the downregulation of HOTAIR promotes autophagy and increases imatinib sensitivity in GIST cells in vivo.

## Discussion

During the past decade, GISTs have emerged as major foci of molecularly targeted therapies for solid tumors^5,6^. Recent population-based studies suggest that GISTs are more common than previously appreciated^6^. The incidence of GIST was 14.5 per million residents, with the highest incidence being observed in elderly patients, and there was no sex difference^10^. With the introduction of imatinib or other tyrosine kinase inhibitors, there has been a significant increase in the GIST survival rate^23^. However, the development of resistance to imatinib over time is common^24^. Despite imatinib being generally well tolerated, it has many side effects, including fatigue, rash, dermatitis, edema, or headache^9,23^. Secondary resistance to imatinib is seen in about half of patients after 2 years of therapy, manifesting as disease progression^25^. Considering the complexity of imatinib-resistant mechanisms, it is essential to perform more studies to discover alternative pathways in imatinib-resistant GISTs.

Many lncRNAs are associated with drug sensitivity. For instance, it was reported that lncRNA CCAT1 is closely related to the drug sensitivity of esophageal cancer^26^. Both our previous study and the present study demonstrated the importance of lncRNA HOTAIR in GISTs. Our study revealed that HOTAIR was upregulated in GIST cells after imatinib treatment. HOTAIR is a prime example of an oncogenic lncRNA, and its elevated expression was first discovered in breast cancer^13^. Furthermore, HOTAIR has been reported to be associated with drug sensitivity in many cancers^12,27^, such as lung cancer and ovarian cancer. However, the overall biological function and the underlying molecular mechanisms of HOTAIR in GISTs remain unclear.

Therefore, we investigated the function of HOTAIR in GISTs. First, we found that HOTAIR was upregulated in both recurrent GISTs and imatinib-treated GIST cells. Additionally, in vitro assays showed that HOTAIR decreased the response of sensitized GIST cells to imatinib treatment. The potential role of autophagy in imatinib resistance was highlighted after transfection of cells with siHOTAIR or pEX-3-HOTAIR. The present study describes mechanistic insights into the implication of autophagy-regulating drug resistance. The mechanisms that enhanced chemoresistance via HOTAIR upregulation varied from protecting GIST cells from apoptosis to the induction of autophagy. Taken together, our results demonstrated that HOTAIR activates autophagy and promotes the imatinib resistance in GIST cells.

Accumulating research has established that many lncRNAs affect other noncoding RNAs, especially miRNAs, and miRNAs may have effects on the regulation of lncRNAs^28,29^. Furthermore, recent studies reported a close relationship between HOTAIR and miR-130a, and miR-130a regulates chemoresistance by influencing autophagy^17,21,30^, which was also confirmed in the present study. Given that miR-130a regulates chemoresistance dependent on autophagy, we further revealed that miR-130a affects autophagy by downregulating *ATG2B*. miRNAs are important regulators of cancer through modulating post-transcriptional protein synthesis by binding to complementary sequences in the 3′-UTR of mRNAs and inhibiting their translation^21^. Consistently, miR‐130a‐3p has a binding site for the 3′-UTR of *ATG2B*, encoding an autophagy-related protein, and inhibited its translation to suppress autophagy. Additionally, we found that the upregulation of HOTAIR did not increase drug resistance after knockdown of *ATG2B*. Our findings suggested that HOTAIR regulates chemoresistance in GIST cells through a mechanism involving the miR-130a/*ATG2B* axis.

In summary, we discovered a new function of HOTAIR in regulating autophagy and chemoresistance in GIST cells by targeting miR-130a directly. Thus, HOTAIR might be a new molecular marker in patients with GIST with imatinib resistance. However, miR-130a is not the only target of HOTAIR regulation; therefore, further studies are required to explore the function of HOTAIR in imatinib resistance in GIST cases. In our future research, we will not only investigate the mechanism of HOTAIR in GISTs, but also study other lncRNAs with differential expression in recurrent GIST.

## Supplementary information

Table supplement

Fig. S1 Diagrams of plasmids used to overexpress HOTAIR

Fig. S2 Diagrams of plasmids used in the dual luciferase reporter assays

## Data Availability

The data sets generated and/or analyzed in the current study are reasonably available upon request from the corresponding author.
