# Supplementary material for: LncRNA-HOTAIR activates autophagy and promotes the imatinib resistance of gastrointestinal stromal tumor cells through a mechanism involving the miR-130a/ATG2B pathway
Source: Cell Death Dis. 2021 Apr 6;12(4):367. doi: 10.1038/s41419-021-03650-7 (PMC8024283; doi:10.1038/s41419-021-03650-7)
Supplement: Supplementary file 1 — Table supplement [file 41419_2021_3650_MOESM1_ESM.docx]

Table S1. List of primer sequences used in the present study

| RNA | Forward | Reverse |
| --- | --- | --- |
| miR-130a-3p | GTCAGTGCAATGTTAAAAGGGCAT | CAGTGCGTGTCGTGGAGT |
| HOTAIR | AACCACGCAGAGAAATGCAG | CTCTCTGTACTCCCGTTCCC |
| ACTB | CCATCATGAAGTGTGACG | GCCGATCCACACGGAGTA |
| GAPDH | TCAAGATCATCAGCAATGCC | CGATACCAAAGTTGTCATGGA |
| ATG2B | CGACATGGTTTACTAGGCG | GGTAGCTGGTTCTTCTTAATGTC |

Table S2 List of small interfering RNA (siRNA) sequences used in the present study

|  | sense（5'-3'） | antisense（5'-3'） |
| --- | --- | --- |
| HOTAIR siRNA | CCCAUGGACUCAUAAACAATT | UUGUUUAUGAGUCCAUGGGTT |
| ATG2B siRNA | GCAACUGGUUCUGAGCCUATT | UAGGCUCAGAACCAGUUGCTT |
| hsa-miRNA-130a mimics | CAGUGCAAUGUUAAAAGGGCAU | GCCCUUUUAACAUUGCACUGUU |
| hsa-miRNA-130a inhibitor | AUGCCCUUUUAACAUUGCACUG |  |
