# Supplementary figures and images for: LncRNA-HOTAIR activates autophagy and promotes the imatinib resistance of gastrointestinal stromal tumor cells through a mechanism involving the miR-130a/ATG2B pathway
Source: Cell Death Dis. 2021 Apr 6;12(4):367. doi: 10.1038/s41419-021-03650-7 (PMC8024283; doi:10.1038/s41419-021-03650-7)

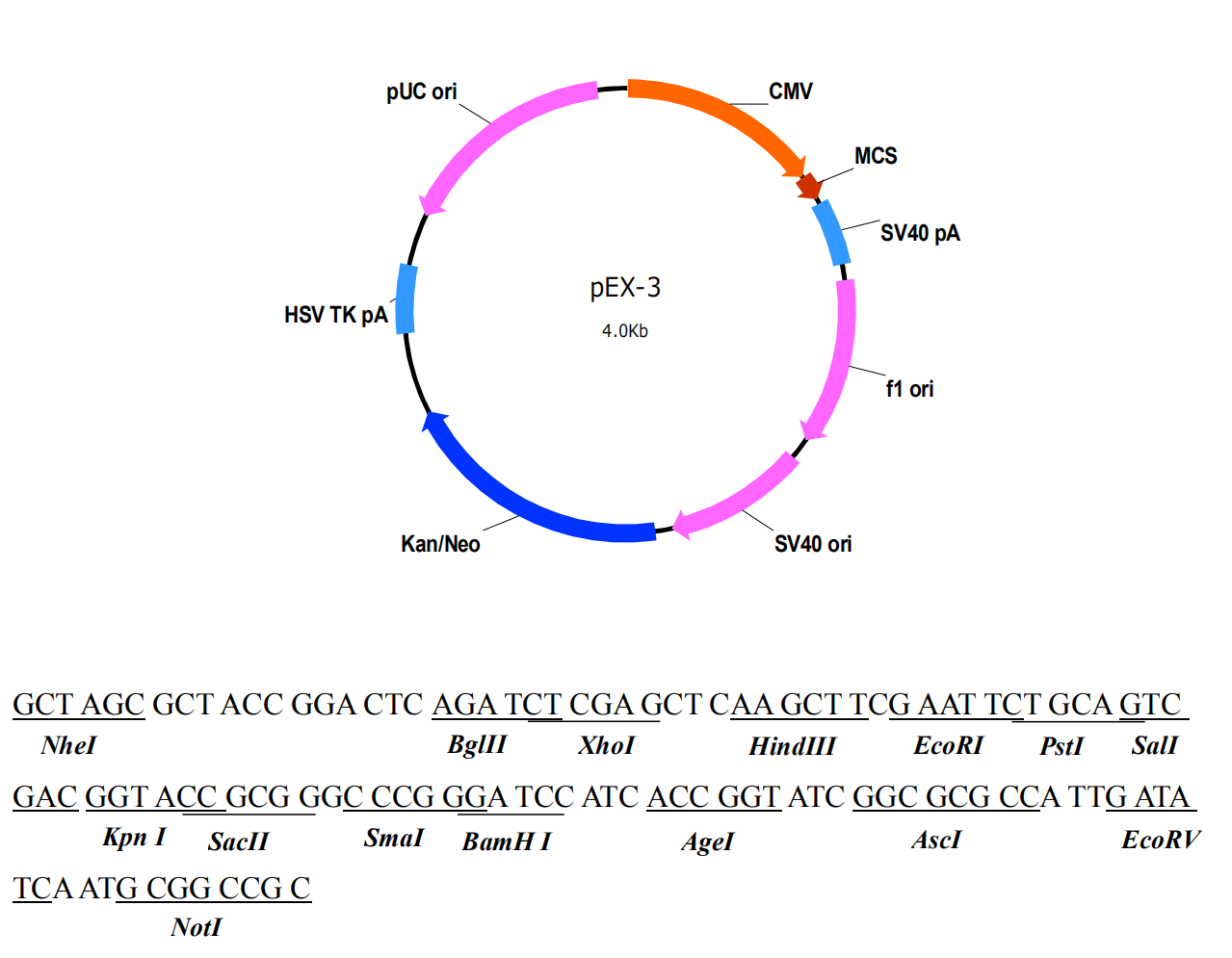

Supplement: Supplementary file 2 — Fig. S1 Diagrams of plasmids used to overexpress HOTAIR [file 41419_2021_3650_MOESM2_ESM.png]

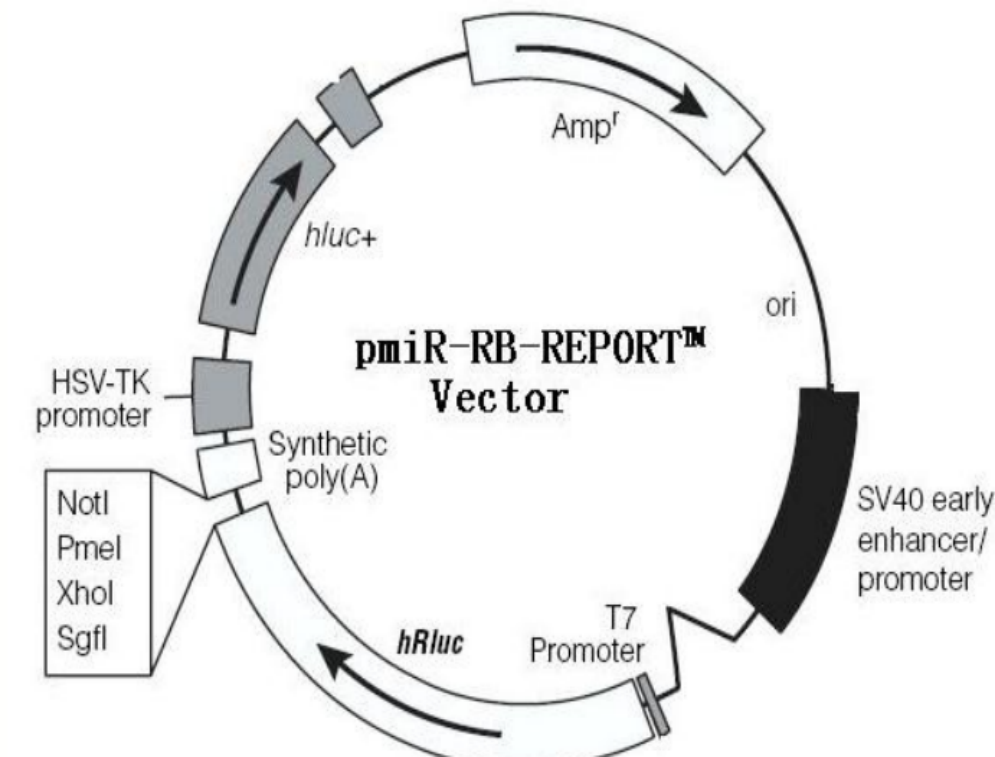

Supplement: Supplementary file 3 — Fig. S2 Diagrams of plasmids used in the dual luciferase reporter assays [file 41419_2021_3650_MOESM3_ESM.png]
